# Effect of a session of intensive exercise with ginseng supplementation on histone H3 protein methylation of skeletal muscle of nonathlete men

**DOI:** 10.1002/mgg3.651

**Published:** 2019-03-28

**Authors:** Ali akbar Naghavi moghadam, Mostafa Shiravand, Sadegh Rezapour, Alireza Khoshdel, Behzad Bazgir, Mahnaz Mardani

**Affiliations:** ^1^ Department of Health, Rehabilitation and Treatment NEZAJA Tehran Iran; ^2^ Faculty of Physical Education and Sport Sciences University of Guilan Gilan Iran; ^3^ Faculty of Medicine Lorestan University of Medical Sciences Khorramabad Iran; ^4^ Faculty of Medicine Aja University of Medical Sciences Tehran Iran; ^5^ Faculty of Life Style, Sport Physiology Research Center Baqiyatallah University of Medical Sciences Tehran Iran; ^6^ Faculty of Health and Nutrition Nutrition Health Research Center, Lorestan University of Medical Sciences Khorramabad Iran

**Keywords:** ginseng supplementation, histone H3 protein (k‐36) methylation, severe periodic exercises

## Abstract

**Purpose:**

The pressure and stress caused by some intense exercises cause changes in histone proteins and gene expression. The aim of this study was to investigate the effect of one session of intensive exercise with supplementation of ginseng, on the methylation of H3K‐36 histone protein in skeletal muscle of young nonathlete men.

**Methods:**

After the approval by the ethics committee, 12 untrained male subjects were randomly assigned to either exercise group (six subjects) or exercise and supplement group. First, from both groups, the muscular sample was taken from the broad‐lateral muscle of the subjects. Immediately after the muscle biopsy, exercise and exercise + supplement groups completed the exercise protocol. During this period, the exercise + supplement group consumed ginseng supplementation and took placebo group. Immediately after exercise, all subjects were retested.

**Results:**

There was no significant increase in histone H3‐k36 protein methylation in the intergroup between exercise + supplementation and exercise. There was a significant difference within the training group but there was no difference in the exercise + supplementation group.

**Conclusion:**

The methylation caused by intense physical pressure, can be reduced by ginseng extract.

## 1 INTRODUCTION

The pressure and stress caused by some intense exercises cause changes in body metabolism. Some studies have shown that the metabolic changes induced by these exercises are similar to some of the hormonal changes caused by some diseases and injuries caused by surgeries, infections, and traumas (Chatzinikolaou et al., 2010). Some of these changes are also attributed to epigenetic changes. One of these changes can be in gene expression (Ntanasis‐Stathopoulos, Tzanninis, Philippou, & Koutsilieris, 2013). Due to its crucial role in the genomic stability maintenance, histone methylation has been assumed to control DNA repair. Histone methylation facilitates the localization of 53BP1 to a DNA double‐strand break (DSB) during repair of homologous recombination (Peng & Karpen, 2009). Changes in transcription of histone proteins play a key role in gene expression and changes in the structure of chromatin (Kouzarides, 2007; Li, Carey, & Workman, 2007). By recognizing the properties of histone proteins, it has been shown that differences in size, amino acids, especially lysine and arginine, and finally solubility are different. At the beginning of the discovery of the histones, they gave different names, but today their naming is H4, H3, H2B, H2A, and H1 (Luger, Mäder, Richmond, Sargent, & Richmond, 1997). Acetylation, deacetylation, and methylation of histones, especially in H3 and H4 histones, have been extensively studied among histone changes. Histone acetylation by the enhancement of histone acetyltransferase (HAT) enzymes will increase digestion, and their deacetylation by HDACs in cases will reduce the number of copies (Clayton, Hazzalin, & Mahadevan, 2006; Forsberg & Bresnick, 2001; Wade, 2001). Meanwhile, methylation by the methyl‐transferase (HMTs) enzyme, depending on the type and position of the methylated amino acids, disable the duplication and also activate the transcription in some genes (Bernstein, Meissner, & Lander, 2007; Pryde et al., 2009).

Studies have shown that athletic activities cause changes in the nucleus of cell by changes in histone proteins, which increase and decrease the gene expression after exercise (McGee & Hargreaves, 2011). It seems that changes in histone proteins play an important role in regulating gene expression and increasing the volume of muscle fibers, their shape, and oxidative capacity. Changes in histone proteins increase the expression of oxidative genes by using calcium‐dependent mechanisms involving kinases (McGee & Hargreaves, 2011). Exercise is one of the important factors for activating some of these kinases in skeletal muscle. Some of these kinases are considered as factors for increasing or decreasing the process of changes in histone proteins and gene expression (McGee & Hargreaves, 2011). In most human studies, it has been shown that changes in histone proteins in skeletal muscle can play a very important role in controlling the gene expression after exercise (Potthoff et al., 2007). For example, Mahoney, Parise, Melov, Safdar, and Tarnopolsky (2005 **)** found that regular physical activity would change the histone proteins and cause the expression of some genes.

One study found that acute and intense gymnastics would increase the H3 K36 stagnation immediately after these activities, but the H3k9 and H3k14 staining would remain unchanged (Saleem & Safdar, 2010). Another study by Harvey et al., 2004 found that immediately after exercise, the protein acetylation of the histone H3 skeletal muscle would increase (Hargreaves, Horng, & Medzhitov, 2009). Sun et al., 2008, in a study on human skeletal muscle, found that aerobic exercise caused changes in HDAC and IIa (McGee, Fairlie, Garnham, & Hargreaves, 2009). A short period of regular physical activity can cause genome hyper‐methylation within the muscle cells. This means that many regulatory genes can be transformed into pathways such as regeneration and muscle growth. Exercise intensity is directly related to the amount of promoter de‐methylation, so intense physical activity activates more genes (Ntanasis‐Stathopoulos et al., 2013).

In addition, nutrition is one of the most important factors in gene expression changes (Han et al., 2005). Today, most people have brought ginseng supplements to increase their physical fitness and improve their health, either as a supplement to exercise or as a supplement to herbal supplements. Ginseng is grown in different parts of the world, including North America, Korea, China, and Siberia (Siegel, 1979). In some studies, it has been determined that consuming ginseng root supplement can act as a contributing factor to the expression of genes that prevent the effects of physical pressure (Han et al., 2005). In a study by Han et al. (2005) on the effect of ginseng supplement on mRNA and TGF‐β1 gene as well as antioxidant activity, it was found that ginseng supplement increased the antioxidant property and the positive effect of TGF‐β mRNA reduces the damage to inflammatory cytokines. Another study found that the use of ginsenosides reduced TNF‐α expression and reduced arthritis (Kim et al., 2007). Although studies have been conducted on the effects of exercise on epigenetic changes, however, they have been limited. Our goal in this study was to determine whether the physical stress associated with short‐term intense exercise alone and with the use of ginseng supplement will alter the methylation of the histone H3 k36 protein of nonathlete subjects.

## 2 MATERIALS AND METHODS

After approval by the Ethics Committee of the Army Medical University of the Islamic Republic of Iran, 12 non‐trained male subjects with mean age of 24.25 ± 2.25, with height 176.2 ± 5.55, weight 86.5 ± 9.15 and BMI 35.4 ± 28.18 out of 15 people were selected randomly, six in the exercise group, and six in the exercise + supplement group. Anthropometric variables were measured after medical confirmation by a specialist physician, then from both groups, fine needle injection (FNI) needle was applied to the skin from the broader muscle of the subjects and deepened by specialist doctor. Before the needle enter the sampling site, it was washed, shaved, and then 1–2 mg of lidocaine, 1% (blistering) was injected into the skin subcutaneous tissue and subcutaneous tissue fascia. Exercise was conducted with complete care to prevent unwanted material from reaching muscle tissue. The sampling needle is about 15 cm above the kneecap, passing through the skin and fascia, imported muscle tissue. This method was so quick that its scar was almost invisible, and subjects did not have to restrict their function. The tissue was harvested at about 50 mg immediately after sampling in formalin solution and transferred to the laboratory (Hayot et al., 2005). Immediately after the muscle biopsy, exercise and exercise + supplement groups completed the exercise protocol.

## 3 PRACTICE PROTOCOL IMPLEMENTATION METHOD

### 3.1 Familiarization meeting

People got acquainted with the practice protocol 1 week before the test. After general and specific warm‐up, the subjects were trained first with low intensity and then severely determined according to the training protocol of this study. In this research, random test was used as a training protocol. This test is one of the most important tests in sport science. It is a family test of Bingation tests such as Wingate or Sargent jump in 1  minute, and it can be used to evaluate important anaerobic factors in athletes (Chakravarty et al., 2008; Zacharogiannis, Paradisis, & Tziortzis, 2004). In some studies, similar tests (Wingate) have been used as training protocols (Saleem & Safdar, 2010). The test was conducted in such a way that a 35 m distance was selected on both sides of which there was enough free space. The subjects then warmed‐up their bodies for 7–10 min. The subjects stood at the starting line, as previously identified, and covering a distance of 35 m, at full speed. At the end of the distance, the athletes rest for 10 s. After 10 s of rest, subjects quickly ran the distance of 35 m at full speed, again. The subjects travelled six times covering 35 m long, and at each stage, the rest time of the subjects was recorded by two referees to record the time of rest (Chakravarty et al., 2008; Zacharogiannis et al., 2004). The German pulse oximeter was used to ensure that the subjects were practicing with maximum heart rate. Immediately after rehearsal, all subjects were subjected to muscle sampling and the muscle samples were transferred to the 10% formalin solution in the laboratory. The immunohistochemistry method and histone H3 (dimethyl k36) monoclonal antibody zellbiogie from Germany were used to evaluate the changes in histone. In this study, 1 hr before completing the exercise protocol, the exercise + supplement group was given a supplementation of ginseng at a dose of 500 mg (CİVAN, KEÇECİ, & ÇAKMAKÇI, 2010).

### 3.2 Immunohistochemistry

#### 3.2.1 Preparation of 4% paraformaldehyde

Twenty‐grams of paraformaldehyde (Merck, Germany) was added to 500 cc distilled water. The hot plate was set to 60°C. In the next step, the solution was added to the desired solution (NaOH) to reach a solution pH of 7.2–7.4. In the final step, a PBS tablet was added to the solution.

#### 3.2.2 Method of doing work

Samples were washed with PBS in four steps and at 5 min intervals. In order to restore antigenicity on the samples, for 30 min, 2N hydrochloric acid was poured. In order to neutralize the acid, borate buffer was added for 5 min. The cells were washed with PBS. For the permeability of the membrane, 0.3% triton was used for 30 min. In the next step, diluted antibody (1–100) with PBS was added to the sample. After creating a wet environment to prevent tissue drying, the samples were placed in a refrigerator overnight at a temperature of 2–8°C. The next day, containers containing tissue were removed from the refrigerator and were washed four times with PBS for 5 min each time. In the next step, the antibodies were added to the samples at a dilution of 1 to 200, and then the muscle samples were incubated at 37°C for 1 hr 30 min in the dark. Subsequently, the samples were transferred from the incubator to the dark room and then washed four times, then PI was added, and PBS was poured onto the samples. In the final step, for confirmation of markers, samples were imaged by Olympus Fluorescent Microscope with a 200 mm lens. Analysis of methylated proteins image was done by image J software.

### 3.3 Statistical analysis

In this study, *t* test was used to test the differences between groups and independent *t* test was used to compare intragroup differences. The significance level for all tests was considered *p* ≤ 0.05. All of these operations were performed using SPSS software and EXCEL software.

## 4 RESULTS

**Figure 1:**
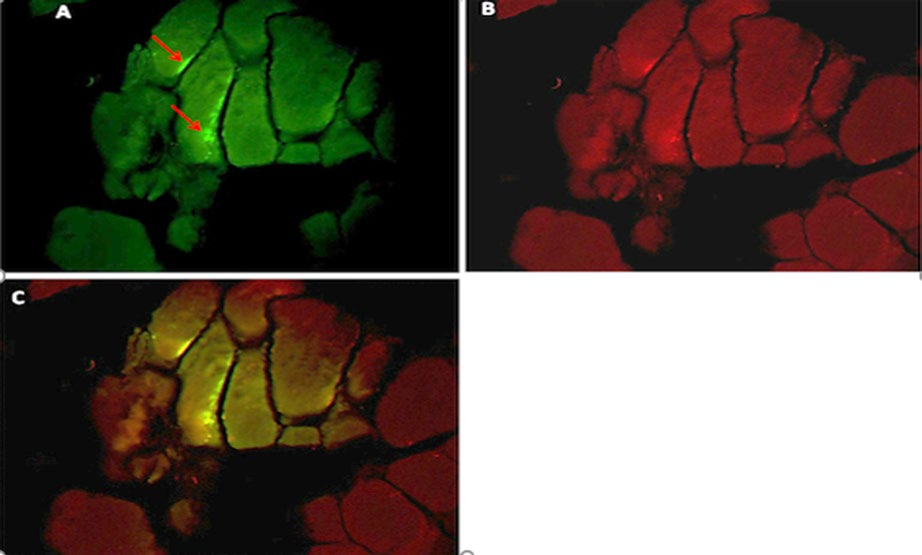
A cross‐sectional view of muscle tissue and percentage of methylation before exercise protocols in the training group

**Figure 2:**
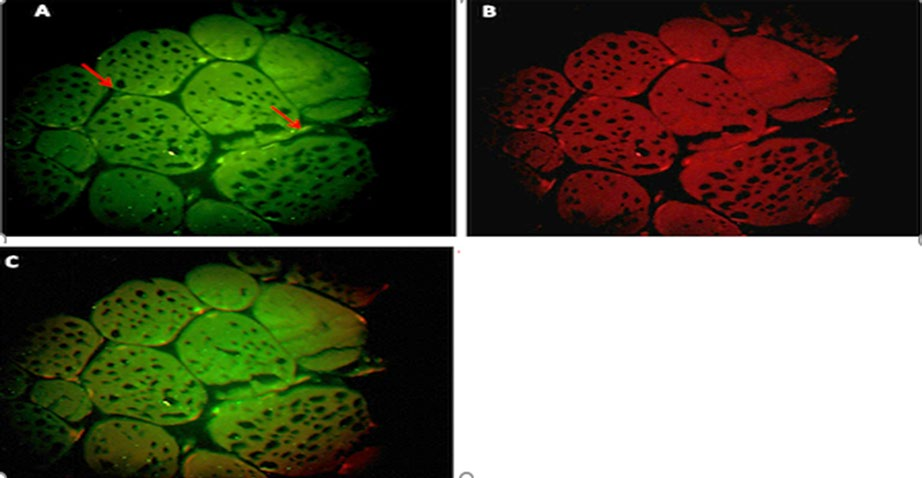
A cross‐sectional view of muscle tissue and percentage of methylation after training protocols in the training group

**Figure 3:**
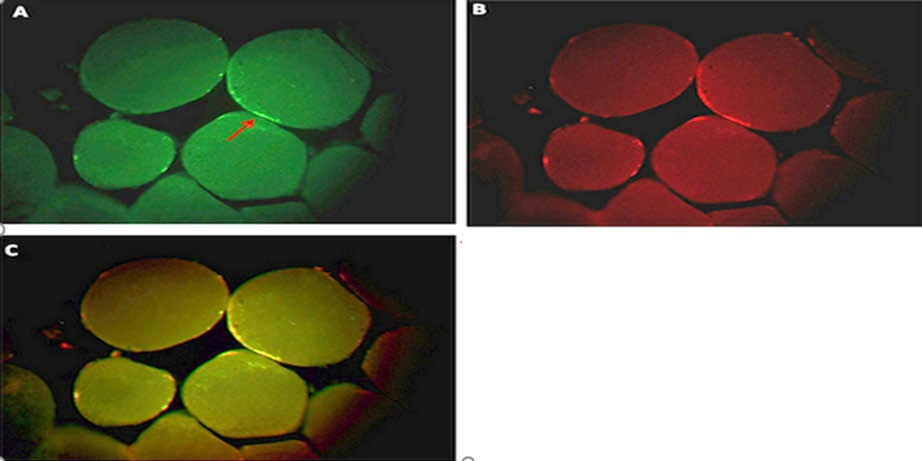
An image of the cross‐sectional muscle cross‐section and the percentage of methylation before exercise protocols in the exercise + supplement group

**Figure 4:**
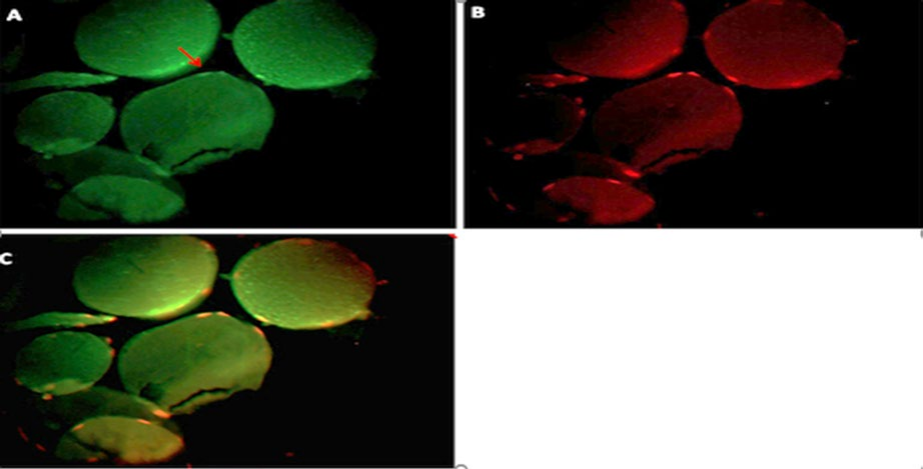
An image of the transverse sectional area of the muscle tissue and the percentage of methylation after the exercise protocol in the exercise + supplement group

The statistical information related to the anthropometric characteristics of the subjects before and after the research protocol is shown in Table 1. The results of the independent *t* test and correlated T is shown in Figure 1, the cross‐sectional sample from the broader muscle tissue and the percentage of methylation before and after the training in the training group is shown in Figures 1 and 2. The cross‐sectional samples of broad‐banded muscle tissue and percentage of methylation before and after training in the training + supplementation group are shown in Figures 3 and 4.

**Table 1:** Physical and anthropometric characteristics of subjects (mean ± *SD*)

Mean ± *SD*		
Groups/Variables	Exercise	Exercise + Supplements
Age	24 ± 2.9	24.3 ± 1.6
Height	172.7 ± 6.5	179.7 ± 5.6
Weight	84.8 ± 8.9	87.3 ± 9.4
Body mass index	28.4 ± 1.6	27.9 ± 7.1

In Figure 1, the percentage of histone H3‐k36 protein methylation before training in the training group was 2.38%, whereas in Figure 2, the same subject after exercise has increased to 24.25%.

As shown in Figure 3, the percentage of methylation of histone H3‐k36 protein before exercise was 1.31% in the exercise group + supplementation group, whereas in Figure 4, after exercise of the same subject it is 1.15%, which has not changed abruptly. Methylation was the same in most other samples in this group.

**Figure 5:**
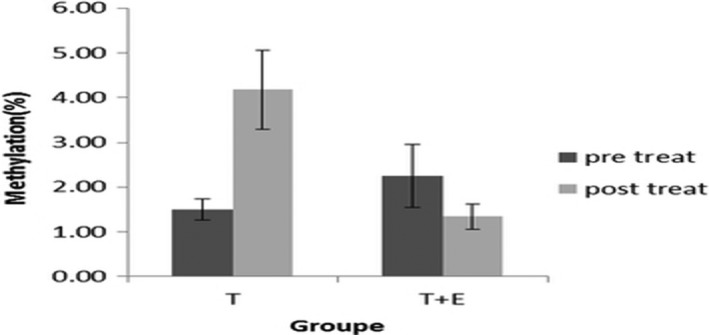
Methylation levels before and after exercise in exercise and exercise and supplementation groups *Significant difference between pretest and posttest at the level of *p* ≤ 0.05

In Figure 5, there was no significant difference between the two groups after exercise. There was a significant difference in intragroup and in exercise group, but in the exercise and supplementation group of methylation, after exercise, the difference was not significantly different from that of the pre‐workout and slightly decreased.

## 5 DISCUSSION AND CONCLUSION

In this study, one session of intense periodic exercises did not significantly differ in the skeletal muscle methylation of histone H3‐K36 between the exercise and exercise + supplement groups. However, comparison within the exercise group shows a severe periodic exercise session, with a significant difference in the methylation of histone H3‐K36 protein, but these exercises, along with ginseng supplementation, did not have a significant effect on the changes in this protein.

So far, there has been no study on the effects of short and severe training on histone H3‐K36 protein methylation, but studies on the effects of these exercises and aerobic exercises have been carried out on other changes in histone proteins. For example, Mahoney et al., (Mahoney et al., 2005) found that regular physical activity would modify histone proteins and cause the expression of some genes. Hargreaves et al., (Hargreaves et al., 2009) found that immediately after exercise, the acetylation protein of the histone H_3_ skeletal muscle would increase. Sean et al., (McGee et al., 2009) in a study on human skeletal muscle, found that aerobic exercise caused changes in HDAC and IIa. In another study conducted by Saleem and Safdar (2010), short and severe exercises in untrained people increased acetylation significantly in histone H3‐K36 proteins. In the above studies, acetylation and deacetylation of histone proteins have been considered, but in this study the methylation of histone proteins has been considered. In general, histone acetylation will increase transcriptional activation in some genes (McGee et al., 2009). But methylation of histone proteins may have different effects on gene expression depending on the number and type of amino acid methylation of these proteins. For example, methylation of lysine in positions of H4k20, H3K27, and H3K9 leads to gene mutation, but methylated lysine's in the position of H3K79, H3K36, and H3k4 are associated with the activation of gene expression (Nguyen & Zhang, 2011; Pryde et al., 2009; Schotta et al., 2004). In some studies, methylation of histone H3 K36 proteins has been shown to protect and enhance duplication through RNA Pol II. It has been shown in this study that histone methylation in the H3K36 position is essential for the conservation and growth of eukaryotic cells (Morris et al., 2005).

With regard to the above, it can be estimated that the result of this study, which focused on methylation of histone H3 protein, can be ascribed to the results of research by Hi et al., which, in terms of the intensity and duration of exercise prototype, as well as type Lysine (Lysine H3 number 36) is similar in the training group (Saleem & Safdar, 2010).

In this study, we used Hibern and the Wingat exercise protocol, and the subjects were not trained. Most importantly, one of the continuous variables studied was histone H3 K36 protein, which, in terms of the level of readiness of the subjects (nonathlete young male), for the sport test (Wingate test), which is a family test used in this study (Test Rast), and in terms of protein type (histone H3 lysine number 36), this study is similar to that in the training group. But in the other studies mentioned above, both the exercise protocol and the type of histone proteins are different from those in the training group.

In addition, in the present study, it was found that periodic intensive exercises with ginseng supplementation did not significantly differ in the methylation of histone H3 k‐36 protein. Similar to histone methylation in the training group, no studies have been done in this field, but studies have been done on the use of complementary ginseng, its antioxidant properties and the effect of the supplementation on mRNA changes in some genes. For example, in a study by Han et al. (2005), it was found that supplementation with ginseng would increase the antioxidant property and the positive effect of TGF‐β1 gene mRNA and would reduce the damage in inflammatory cytokines (Han et al., 2005). Another study by Huang et al., concluded that the use of this supplement would reduce TNF expression in humans (Huang, Yamashiki, Nakatani, Nobori, & Mase, 2001). In various studies, it has been found that the use of ginseng supplementation would increase blood oxygen and also increases strength and speed of muscle (Grandhi, Mujumdar, & Patwardhan, 1994; Pieralisi, Ripari, & Vecchiet, 1991; Wasuntarawat et al., 2010). For example, in a test that was performed on 24 women, it was shown that supplementation for 8 weeks reduces fatigue and increases the power turns (Grandhi et al., 1994).

In a double‐blind study, it was shown that ginseng supplementation could increase the maximum velocity (Wasuntarawat et al., 2010). In another study, it was found that ginseng supplementation and aerobic exercise did not increase the bleeding capacity of untrained people (Pieralisi et al., 1991). In the present study, increase in speed and strength, as well as increase in the maximum oxygen consumption, affect epigenetic changes were not determined.

From Figure 5 of the present study, there was no significant difference in the methylation of histone H3‐k36 in the comparison between the two groups of exercise and exercise + supplementation. Intragroup comparison did not increase the H3‐K36 histone methylation supplementation in the exercise group, and even slightly decreased. In comparing the outcome of this group with the result of the exercise alone, we can estimate that the intake of supplements, together with periodic exercise, has reduced the methylation of histone H3 protein in lysine number 36. But this decrease was low and did not result in a significant difference between the intergroup and the intragroup, which may be due to the effects of reducing physical pressure and the effect of reducing oxidative indices by increasing some of the physiological indices.

In conclusion, the positive results from the use of ginseng supplement, increases positive indexes and reduced physical stress. This in‐turn reduces the need for histone H3K‐36 methylation, or because of single‐session consumption, the result would be different when taken for long term. The methylation caused by intense physical pressure, however, can be reduced by ginseng extract.

## CONFLICT OF INTEREST

The authors deny any conflict of interest in any terms or by any means during the study. All fees are provided form research center fund and disbursed accordingly.

## AUTHOR CONTRIBUTIONS

SR and MM: Planned the study, wrote the protocol, collected the data, drafted the manuscript, and accepted the final draft; Ali akbar Naghavi moghadam, Mostafa Shiravand, Ali reza Khoshdel, and Behzad Bazgir: Planned and designed the study, collected the data, analyzed the data, critically revised the draft, and finally approved the manuscript.
